# mRNA Quantification of *NIPBL* Isoforms A and B in Adult and Fetal Human Tissues, and a Potentially Pathological Variant Affecting Only Isoform A in Two Patients with Cornelia de Lange Syndrome

**DOI:** 10.3390/ijms18030481

**Published:** 2017-02-23

**Authors:** Beatriz Puisac, María-Esperanza Teresa-Rodrigo, María Hernández-Marcos, Carolina Baquero-Montoya, María-Concepción Gil-Rodríguez, Torkild Visnes, Christopher Bot, Paulino Gómez-Puertas, Frank J. Kaiser, Feliciano J. Ramos, Lena Ström, Juan Pié

**Affiliations:** 1 Unit of Clinical Genetics and Functional Genomics, Department of Pharmacology-Physiology and Paediatrics, School of Medicine, University of Zaragoza, CIBERER-GCV02 and ISS-Aragon, E-50009 Zaragoza, Spain; puisac@unizar.es (B.P.); eteresa@unizar.es (M.-E.T.-R.); mhmarcos@unizar.es (M.H.-M.); mcgil@unizar.es (M.-C.G.-R.); framos@unizar.es (F.J.R.); 2 Department of Pediatrics, Hospital Pablo Tobón Uribe, 05001000 Medellín, Colombia; carobaque@gmail.com; 3 Department of Cell and Molecular Biology, Karolinska Institute, SE-17177 Stockholm, Sweden; Torkild.Visnes@ki.se (T.V.); Christopher.Bot@ki.se (C.B.); Lena.Strom@ki.se (L.S.); 4 Molecular Modelling Group, Center of Molecular Biology “Severo Ochoa” (CSIC-UAM), Cantoblanco, E-28049 Madrid, Spain; pagomez@cbm.csic.es; 5 Section for Functional Genetics at the Institute of Human Genetics, University of Lübeck, D-23538 Lübeck, Germany; frank.kaiser@uk-sh.de; 6 Unit of Clinical Genetics, Department of Paediatrics, Hospital Clínico Universitario “Lozano Blesa”, CIBERER-GCV02 and ISS-Aragon, E-50009 Zaragoza, Spain

**Keywords:** Cornelia de Lange syndrome, *NIPBL* isoform A, *NIPBL* isoform B, splicing variants, mRNA, adult tissues, fetal tissues, *NIPBL* pathological variant

## Abstract

Cornelia de Lange syndrome (CdLS) is a congenital developmental disorder characterized by craniofacial dysmorphia, growth retardation, limb malformations, and intellectual disability. Approximately 60% of patients with CdLS carry a recognizable pathological variant in the *NIPBL* gene, of which two isoforms, A and B, have been identified, and which only differ in the C-terminal segment. In this work, we describe the distribution pattern of the isoforms A and B mRNAs in tissues of adult and fetal origin, by qPCR (quantitative polymerase chain reaction). Our results show a higher gene expression of the isoform A, even though both seem to have the same tissue distribution. Interestingly, the expression in fetal tissues is higher than that of adults, especially in brain and skeletal muscle. Curiously, the study of fibroblasts of two siblings with a mild CdLS phenotype and a pathological variant specific of the isoform A of *NIPBL* (c.8387A > G; P.Tyr2796Cys), showed a similar reduction in both isoforms, and a normal sensitivity to DNA damage. Overall, these results suggest that the position of the pathological variant at the 3´ end of the *NIPBL* gene affecting only isoform A, is likely to be the cause of the atypical mild phenotype of the two brothers.

## 1. Introduction

Brachmann-de Lange syndrome (BDLS) or Cornelia de Lange syndrome (CdLS, OMIM: 122,470, 300,590, 610,759, 614,701, 300,882) is a rare congenital dysmorphogenic disorder with a high degree of variability in its clinical presentation [1]. The main characteristics are typical craniofacial dysmorphia, growth retardation, upper limb malformations, intellectual disability, hirsutism, and malformation of major organ systems, especially the digestive one [2].

In addition, CdLS shows a very extensive genetic heterogeneity, and pathological variants in *NIPBL* (5p13.1), *SMC1A* (Xp11.2), *SMC3* (10q25), *RAD21* (8q24), and *HDAC8* (Xq13.1) genes, have been described [3,4,5,6,7,8]. All of these genes code for regulatory or structural proteins of the cohesin complex, essential for the proper function of several biological processes, such as chromosome segregation, DNA repair, gene expression regulation, and chromatin remodeling, among others [9]. Pathological variants in the cohesin regulator *NIPBL* are present in approximately 60% of classical CdLS patients [3,4]. In general, more damaging pathological variants (nonsense, frameshift) cause a more severe phenotype than less damaging pathological variants (missense) [10]. The *NIPBL* gene contains 47 exons and, although several transcripts have been identified in peripheral blood leukocytes [11], the main and largest size splicing variants are isoforms A and B. Both isoforms are conserved in vertebrates and have identical aminoacids from 1 to 2683, while the C-terminal ends are different [4]. In the isoform A, exons 2–47 encode for the complete protein that has 2804 aminoacids. The alternative, isoform B, does not include exon 47 and ends in an expanded variant of exon 46, with a total of 2697 aminoacids.

Although previous studies have analyzed the total expression of the *NIPBL* gene by Northern blot [4], this paper reports, for the first time, on the mRNA distribution pattern of the A and B isoforms in different tissues of adult and fetal origin, quantified by quantitative PCR (qPCR). Moreover, we studied two brothers with CdLS and a missense pathological variant that exclusively affected the isoform A of the *NIPBL* gene. In addition, the presence of somatic mosaicism was ruled out and the effect of ionizing radiation on fibroblasts was assessed. Our results showed that these patients have a mild phenotype with less sensitivity to DNA damage, when compared to patients with severe CdLS, caused by mutations affecting both isoforms A and B.

## 2. Results

### 2.1. Distribution of mRNA NIPBL Isoforms A and B in Foetal and Adult Human Tissues

**Figure 1:**
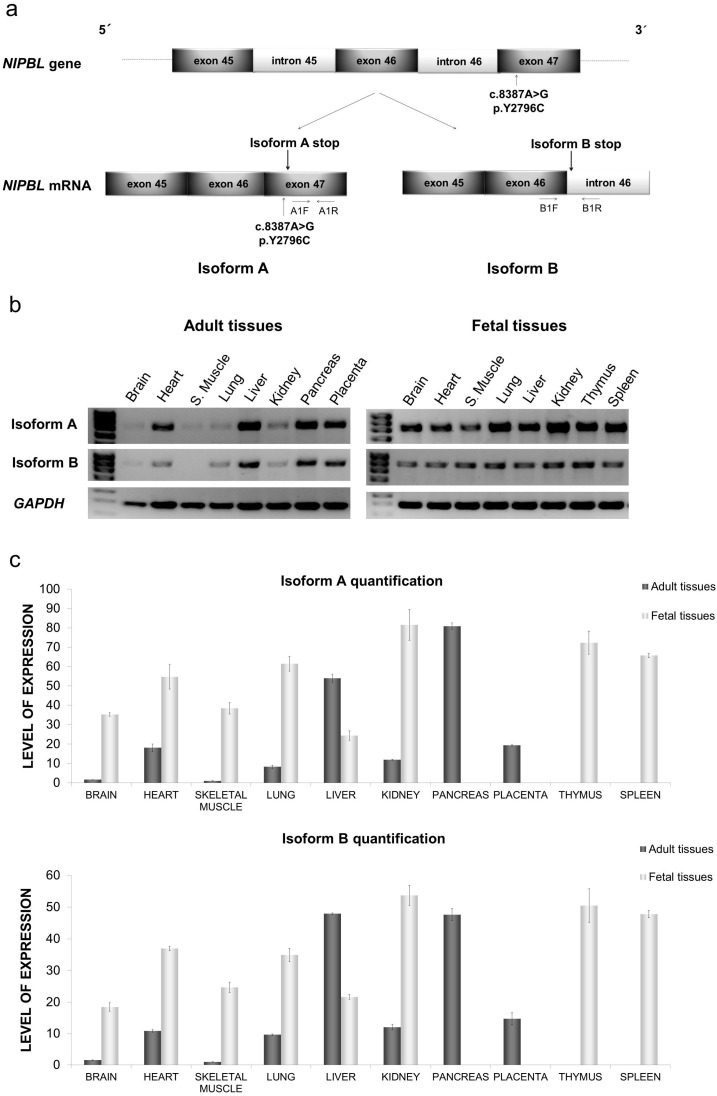
Study of *NIPBL* isoforms A and B in human tissues (**a**) Schematic representation of the 3’ region of the *NIPBL* gene and the resulting mRNA *NIPBL* isoforms A and B. The location of primers used for qPCR (quantitative polymerase chain reaction) to detect specific *NIPBL* isoforms A (AF1/AR1) and B (BF1/BR1) are indicated by arrows. The position of the pathological variant in exon 47, exclusive to isoform A, is also indicated; (**b**) RT-PCR (reverse transcription polymerase chain reaction) analysis of mRNA expression of *NIPBL* isoforms A and B. cDNA from normal adult and fetal tissues was used for the specific amplification of *NIPBL* isoforms A or B. Samples were analyzed by agarose gel-electrophoresis and *GAPDH* was used as a loading control; (**c**) Quantitative PCR analysis of mRNAs expression levels of *NIPBL* isoforms A and B in different tissues. Ct values were compared to standardized curves and normalized against *GAPDH*. The mean level of expression in adult skeletal muscle tissue was considered as 1. All the tissues (adult and fetal) are expressed as a relative fold. Results are presented as means + standard deviation, *n* = 3.

In a first approximation by RT-PCR, the specific amplification of both isoforms A and B from the cDNA of different human tissues showed a similar tissue distribution pattern, although the levels of isoform A were always higher than those of B (Figure 1b). Both isoforms appeared to be expressed in a higher quantity and more homogeneously in fetal tissues, than in adult tissues.

### 2.2. Quantitative mRNA Analysis of Isoforms A and B in Foetal and Adult Human Tissues

The mRNA levels of the *NIPBL* isoforms were accurately quantified by qPCR, using specific primers for each of them (Figure 1a,c). For both isoforms A and B, the value of 1 was assigned to the reference tissue of minimal expression, i.e., the adult skeletal muscle. The expression in all other tissues (adult and fetal) was then expressed in relation to this reference value.

The expression levels of isoform A in fetal tissues were higher than those in adult tissue, except for the liver, where the adult expression was higher (54 vs. 24 times higher than the reference level) (Figure 1c). The mRNA levels of isoform A in the fetal skeletal muscle and brain were 38 and 35 times higher than those in adult tissues, respectively (Figure 1c).

The highest expression of isoform A was obtained in the fetal kidney and adult pancreas (80 times higher than the reference tissue). Significant levels, in the range of 72–54 times higher than the reference, were found in the fetal thymus, spleen, lung, and heart, and the adult liver (Figure 1c).

The mRNA levels of isoform B, although clearly lower than that of isoform A, follow the same pattern of distribution (Figure 1c), as in Figure 1b. mRNA levels were higher in fetal tissues than in those of adults, except for the liver (47 times in adult/21 times in fetal), whereas in the fetal skeletal muscle and brain, they were 24 and 18 times higher than in adult tissue, respectively (Figure 1c). Peak levels were found in the fetal kidney, spleen, and thymus, and the adult pancreas and liver (53–47-fold), and minimum levels were found in adult skeletal muscle and the brain (Figure 1c).

### 2.3. Structure Prediction of the C-Terminal Segment of the Isoforms A and B of the NIPBL Gene

DP-Bind server (http://lcg.rit.albany.edu/dp-bind), a system for the sequence-based prediction of DNA-binding residues in proteins, suggested that the C-terminal segment of *NIPBL* isoform A (residues 2653–2804) is likely to be involved in DNA binding. A RaptorX system [12] was used to obtain a putative structure for this segment. The system proposed five different templates for the C-terminal residues 2541–2804 of isoform A, all of which had medium to medium-low internal scores for confidence. The structure with the better scores (*p*-value = 1.4 × 10^−2^, Score = 55, uGDT/GDT = 27/16 and uSeqID/SeqID = 16/9) corresponded to the DNA-binding domain of human cytomegalovirus DNA polymerase (Protein Data Bank entry = 3kd1E). Using this structure as a template, a 3D model was generated for the C-terminal domain of *NIPBL* isoform A (Figure S1). In brief, and according to this model, the aminoacidic sequence present in isoform A and absent in isoform B (residues 2683–2804), is predicted to form the domain that directly interacts with the putative DNA molecule, exhibiting a positive charge in the protein surface that is complementary to the negative charge of DNA. Conversely, *NIPBL* isoform B lacks this DNA-binding domain.

### 2.4. Clinical Description

The patients studied here are two stepbrothers of Spanish origin. The mother had a phenotype suggestive of mild CdLS. The mother had withdrawn the guardianship of her children and did not give her consent for the genetic study of herself. For this reason, she could not be included in the results.

#### 2.4.1. Patient 1

**Figure 2:**
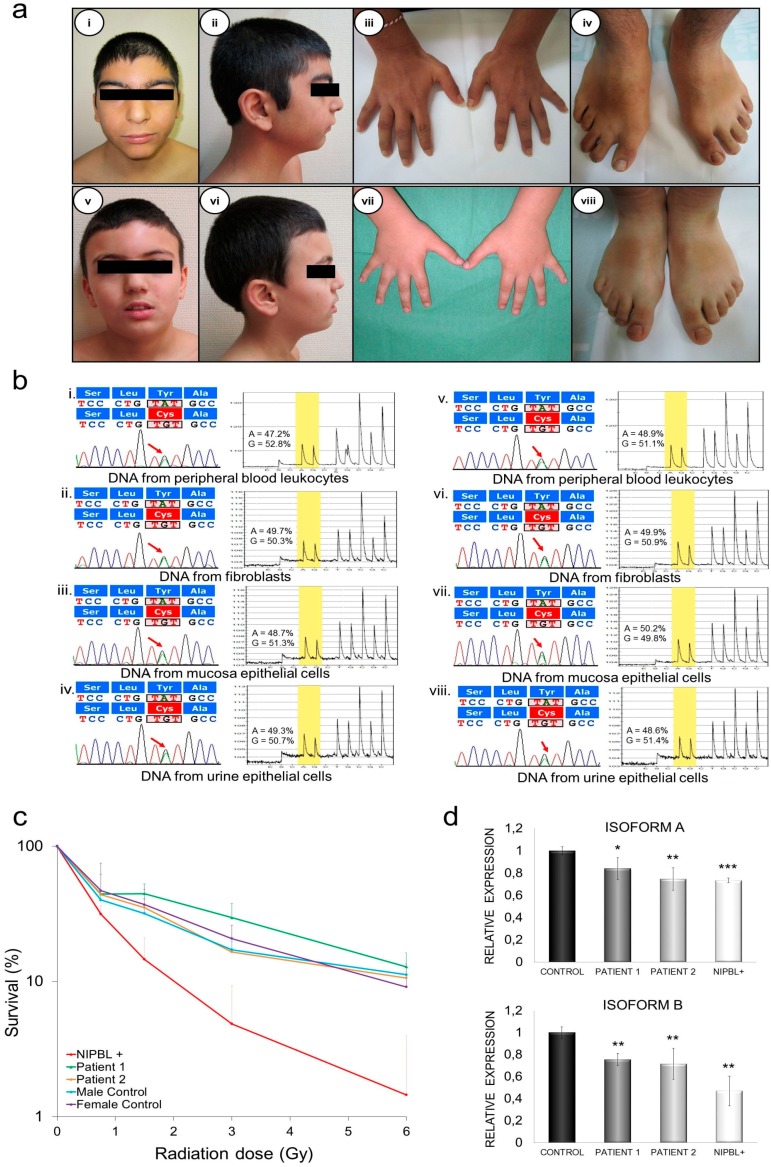
Overview of the phenotype and molecular findings of the two patients (**a**) Phenotype of patient 1 and patient 2. Frontal and lateral view of each patient (i, v, ii, vi), hands (iii, vii) and feet (iv, viii); (**b**) Detection of the pathological variant c.8387A > G (p.Tyr2796Cys) in patient 1 and patient 2 on DNA from peripheral blood leukocytes (i–v), fibroblasts (ii–vi), epithelial cells from oral mucosa (iii–vii), and from urinary tract (iv–viii). For each tissue, the chromatogram corresponding to Sanger sequencing and pyrosequencing quantification are shown; the red arrows indicate the nucleotide substitution position. In pyrograms, A versus G peak are yellow shading (**c**) Evaluation of the sensitivity to radiation referred to as a percentage of surviving cells vs. radiation dose. Each experiment was performed at least twice in triplicate. Note the similar pattern shown for patients P1 and P2, and healthy controls, and the different pattern shown for a *NIPBL*+ patient; (**d**) Quantitative PCR analysis of mRNA’s expression levels of *NIPBL* isoforms A and B from fibroblasts of patients and a control * *p* < 0.05, ** *p* < 0.01, *** *p* < 0.001.

Patient 1 is a 14-year-old male with a clinical diagnosis of CdLS, whose anthropometric data at birth and at his last evaluation are shown in Table 1. He had microcephaly, a low hair implantation line, arched eyebrows, synophrys, long and thin eyelashes, an atypical wide nose with a broad nasal bridge and tip, a long philtrum, a fine upper lip, a high palate, and microretrognatia (Figure 2a). He had normal hearing and no ophthalmological, gastrointestinal, or cardiac anomalies. Furthermore, he had a history of several low respiratory tract infections. He had pectum excavatum, scoliosis, flat feet, bilateral brachyclinodactyly of the fifth finger, bilateral 2–3 toe syndactyly, and 4th toe brachydactyly (Figure 2a, Table 1). Psychometric assessment showed a mild intellectual disability (IQ 44 in WISC-IV) and behavioral problems ADHD (attention deficit hyperactivity disorder), obsessive-compulsive behavior, aggressiveness, and self-injurious behavior) that needed pharmacological treatment. All together, and according to the severity classification criteria established by Kline et al. [2], findings in this patient fit with a mild CdLS spectrum.

#### 2.4.2. Patient 2

Patient 2 is a 10-year-old male, the stepbrother of patient 1, and the third child of his sibship. He had brachymicrocephaly, a shortened biparietal distance, arched eyebrows with synophrys, long and thin eyelashes, maxillar flattening, a wide nose with a broad nasal bridge, low-set and protuberant ears, micrognathia, and a short neck (Figure 2a, Table 1). He had 2–3 syndactyly in both feet (Figure 2a, Table 1). He also had strabismus of the left eye and a heart right bundle branch block with normal cardiac function. He had asthma until the age of three years. He had swallowing problems during the neonatal period, followed by recurrent vomiting and epigastralgia later in life, leading to the suspicion of gastroesophageal reflux disease (GERD) (Table 1). Psychiatric assessment showed a mild intellectual disability (IQ 75 with Raven test) and several behavioral problems (ADHD and self-injurious behavior) that needed pharmacological treatment (Table 1). As seen for his stepbrother, and according to the severity classification criteria established by Kline et al. [2], findings in this patient fit with a mild CdLS spectrum.

### 2.5. Genetic Diagnosis and Pyrosequencing Analysis in Different Tissues

The pathological variant identified in the blood DNA of both patients was c.8387A > G, p.Tyr2796Cys, located at the last exon (E47) of the *NIPBL* gene. This missense pathological variant causes the change of aminoacid Tyrosine 2796 to Cysteine, and it only affects isoform A (Figure 1a). The residue (Y2796) is highly conserved in vertebrates. The Polyphen-2 program [13] predicted a probably damaging effect of this missense pathological variant on the NIPBL protein, with a score of 0.999. The SNPpredict program [14] also considers this pathological variant as deleterious, with an expected accuracy of 87%. The presence of other variants in the *NIPBL* gene, or a mutation in the other causal genes of the syndrome *SMC1A*, *SMC3*, *HDAC8*, and *RAD21*, have been ruled out by sequencing.

On the other hand, pyrosequencing was used for the precise quantification of the wild type and mutant alleles in the genomic DNA of the different tissues (leukocytes, fibroblasts, buccal epithelial cells, and epithelial cells from the urine). In all of the tissues, the expression of one allele against the other was equivalent, with values which were consistently around 50% (Figure 2b).

### 2.6. DNA Damage Sensibility in Patients’ Fibroblasts

A study of the sensitivity to DNA damage was carried out by exposure to increasing doses of gamma radiation to the fibroblasts of the two patients, two healthy control subjects, and one patient with a pathological variant in *NIPBL*, affecting both isoforms A and B, as previously reported [15,16]. The survival of cells was monitored using a colony formation assay. Although there was some variation between the results, patient 1 and patient 2 were no more sensitive to ionizing radiation than the healthy child control cells (*p* > 0.05). In contrast, the fibroblasts from the patient with *NIPBL*+ was easily sensitized by ionizing radiation, even at very low radiation doses (Figure 2c).

### 2.7. Quantification of mRNA NIPBL Isoforms A and B in Patients’ Fibroblasts

The mRNA levels of *NIPBL* isoforms A and B were then assessed for the fibroblasts of patient 1, patient 2, and the same *NIPBL*+ patient in Figure 2c, using qPCR with the primers and strategy described for normal tissues. For each of the isoforms, a 100% value was assigned to the mean expression obtained in fibroblasts from control individuals, and the expression in each patient was reported as a percentage relative to that value. In the patients studied, reduced levels of isoform A were observed when compared to controls: patient 1 (84%, *p* < 0.05), patient 2 (74.6%, *p* < 0.01), and the *NIPBL*+ patient. (73.4%, *p* < 0.001) Isoform B was also reduced in all patients, with the following values: patient 1 (75.5%, *p* < 0.01), patient 2 (71.5%, *p* < 0.01), and the *NIPBL*+ patient (47%, *p* < 0.01) (Figure 2d).

## 3. Discussion

Heterozygous pathological variants in the *NIPBL* gene are the main cause of Cornelia de Lange syndrome, the most frequent cohesinopathy. In order to better understand the pathogenesis of the syndrome, it is necessary to study the functions of the NIPBL protein in greater depth, implicated not only in cohesin loading, but also in the regulation of gene expression during development [17,18]. This variety of functions could be achieved through the generation of alternative transcripts by splicing, one of the most efficient mechanisms of protein diversification [19]. For this reason, we studied the two main splice variants of the *NIPBL* gene, isoforms A and B, in adult and fetal human tissues, and in two patients with CdLS who were harbouring a pathological variant specific for isoform A. Isoforms A and B differ only at the carboxy-terminal end, and thus, isoform A encodes the complete protein, whereas isoform B lacks exon 47 and ends in an expanded form of exon 46 [4]. Although the function of the final segment that differentiates them is unknown, it has been suggested that it interacts with DNA [20]. Our in silico analyses seem to confirm this hypothesis and suggest that the specific segment of isoform A interacts with DNA (Figure S1). However, further studies will be needed to confirm this hypothesis.

Results of qPCR showed that isoform A is present in higher amounts than isoform B, in all the studied tissues. Despite the differences, both variants have an extraordinarily similar pattern of tissue expression (Figure 1b,c), which seems to support a coordinated regulatory mechanism, as has been described for other genes [21,22]. On the other hand, the analysis of the number of transcripts reflects a higher production in fetal tissues, which could be justified by the importance of the cohesins for the regulation of gene expression during embryonic development [9]. Interestingly, brain and skeletal muscle had a very low expression in adult tissues, and this was up to 35 and 38 times lower than in fetal tissues, respectively. Liver tissue is, however, the exception to the rule, with a lower expression in fetal tissue, than in adult tissue. Among all of the tissues analyzed, the highest expression was found in the kidney, thymus, spleen, and fetal lung, as well as in the pancreas and adult liver. These values broadly coincide with the semi-quantitative total expression obtained by Tonkin [4] using Nothern blot, although he obtained lower values for an adult liver. However, there is no apparent direct relationship between the tissues with a higher expression of *NIPBL* and the clinical expressivity of the syndrome, since CdLS patients, in general, do not have a severe involvement of the kidney, liver, or pancreas. However, a better understanding of the expression of these isoforms in specific tissues could be a first step in the search for possible therapeutic options for CdLS, based on the modulation of splicing.

Here, we also report on two stepbrothers with a new missense c.8387A > G pathological variant (Tyr2796Cys) located in exon 47, which only affects the isoform A of the *NIPBL* gene. Both patients have a mild phenotype according to the Kline´s severity score. Both show some characteristic facial features of CdLS, but also have a wide nose with a broad nasal bridge and tip, similar to that described in patients who have CdLS with pathological variants in the *HDAC8* gene [23]. The last evaluation at 14 years of patient 1, and at 10 years of patient 2, showed a normal growth development and a mild intellectual disability that reinforces the initial diagnosis of low affectation. In addition, somatic mosaicism was ruled out as the possible cause of the mild phenotype. Studies on tissues of different embryological origin: leukocytes and fibroblasts (mesoderm), oral mucosa (ectoderm), and urinary epithelium (endoderm and ectoderm), showed a percentage of mutated alleles close to 50% (Figure 2b).

Despite its limited clinical information, a study of other pathological variants exclusive to isoform A, described in the LSDB database [24], revealed a tendency to produce mild phenotypes, even in frameshift variants (c.8377C > T, p.Arg2793Term 2; c.8364delT, ex47 p.Val2789PhefsX36), similar to the findings observed in our patients.

It also appears that the mild clinical manifestations of the two stepbrothers correspond to what is observed at the cellular level. Although it is well known that CdLS patients´ cells have an increased sensitivity to DNA damage [16,25], our patients’ fibroblasts behaved the same as controls, i.e., they were significantly less affected by ionizing radiation than a CdLS patient with a pathological variant affecting both isoforms (Figure 2c). This is the first report of patients with a clinical phenotype of CdLS and a mutation in *NIPBL* that do not show sensitivity to DNA damage. It is also possible that the phenotype in these patients was dependent on a quantitatively different expression of both isoforms, since isoform B is not altered by the pathological variant. However, a decrease in isoform A (16% and 25.4% comparing to the control) was observed in both patients, similar to what was observed in isoform B (24.5% and 28.5%) (Figure 2d). These findings suggest that the reduction of expression occurs before mRNA maturation. In the future, quantification of the expression of these isoforms in a large number of patients with distinct pathological variants in *NIPBL*, may be essential for understanding the contribution of each isoform in the phenotype of CdLS patients.

In conclusion, our results indicate that isoform A is more expressed than isoform B, although they display a very similar tissue distribution pattern. In addition, both isoforms are more abundant in foetal tissues than in adult tissues, especially in brain and skeletal muscle, which can be related to the development of these organs. It is also suggested that patients who only have a pathological variant in isoform A, have a milder CdLS clinical phenotype and a lower sensitivity to DNA damage.

## 4. Materials and Methods

### 4.1. Identification of NIPBL Isoforms A and B in Foetal and Adult Human Tissues

cDNA from different foetal and adult human tissues: brain, heart, skeletal muscle, lung, liver, kidney, pancreas (adult), placenta (adult), thymus (foetal), and spleen (foetal), were purchased from Clontech (Mountain View, CA, USA). Using the primers sF43 (5′-ACATTACACTC TCAGTTTCTG-3′), sR47 (5′-ACGTCAACACTTTCATTC-3´), and sR46b (5′-GATAGAGATTCTCTACTTACCCG-3′), we specifically amplified isoform A (sF43/SR47) and isoform B (sF43/SR46b). The identity of all PCR products was confirmed by DNA sequencing, using the “ABI Prism BigDye Terminator Cycle Sequencing v2.0” Kit (Applied Biosystems, Foster City, CA, USA), and was analyzed on an Applied Biosystems 5700 DNA sequencer (Foster City, CA, USA).

### 4.2. Quantification of mRNA NIPBL Isoforms A and B by qPCR in Human Tissues

The quantification of *NIPBL* isoforms A and B was performed for the cDNA of foetal and adult human tissues from normalized commercial panels Human Fetal MTC Panel and Human MTC Panel I, respectively (Clontech Laboratories, Mountain View, CA, USA). Isoform A was amplified using primers *NIPBL* A1F (5′-GGCAGCACAGATGAATGAAAG-3′) and *NIPBL* A1R (5′-CTTGCAATTTGTGGTCGATCTT-3′), and isoform B with primers *NIPBL* B1F (5′-AATACAGCAGCAGAGACAGAAG-3′) and *NIPBL* B1R (5′-CGAAATACTTGACTCCTCCTC-3′). Standardized curves were used to quantify the expression of each isoform. To create the standardized curves, the cDNA of *NIPBL* isoforms A and B were inserted into the pCR-2.1-TOPO vector, according to the manufacturer’s protocol. Plasmids were quantified by spectrophotometric analysis at 260 nm, and standardized curves were based on a 10-fold serial dilution of the different cloned isoforms. The *C*_t_ (Cycle threshold) values of the samples were interpolated to the corresponding standardized curve. Reactions were performed by using a Power SYBR Green Master Mix, following the manufacturer’s recommendations. Each reaction contained 1 µL of cDNA in a 20 µL mixture. In each experiment, control samples were included: (1) a no template control (NTC); and (2) a no reverse transcriptase (-RT) control. The identity of the PCR products and their purity in each sample were controlled after the last amplification cycle by melting curve analysis. The same procedure was used on *GAPDH*, in order to normalize it. All of the samples were quantified in triplicate, and the average values were calculated.

### 4.3. Patients

This study includes two new Spanish patients who met the criteria for CdLS, according to Kline et al. [2]. Ethical recommendations of the Declaration of Helsinki were followed. The patients’ tutor signed the informed consent to participate in the study. To establish comparisons, we also used cells from two anonymous unaffected individuals and from a patient with CdLS, previously reported by us [15,16]. The present study was approved by the Ethics Committee for Clinical Research of Aragon, on February 2013 (reference number: 04/2013).

### 4.4. Genetic Diagnosis and Pyrosequencing Analysis

We collected DNA samples from both patients from four tissues with similar and/or of different embryological origin (mesoderm (peripheral blood leukocytes and fibroblasts), ectoderm (oral mucosa epithelial cells), and endoderm/ectoderm (urinary epithelium), by standardized protocols. The coding regions and flanking intronic sequences of *NIPBL* (exons 2–47), *SMC1A*, *SMC3*, *HDAC8*, and *RAD21* were screened for pathological variants by PCR amplification of blood genomic DNA. The PCR products were purified with USB ExoSAP-IT PCR Product Cleanup (Affymetrix, Santa Clara, CA, USA), following the manufacturer’s instructions, and were sequenced in a ADN3130 Genetic Analyzer (Applied Biosystems). Parental genotypes were screened to assess whether the variant was de novo or inherited. *NIPBL* cDNA was numbered according to the *NIPBL* isoform 1 (GenBank accession number NM 000642). The pathological variant nomenclature was applied following the instructions from the Human Genome Variation Society [26].

Quantification of the relative amount of each allele in DNA samples from blood leukocytes, fibroblasts, oral mucosa epithelial cells, and urinary epithelium, were performed by the pyrosequencing analysis of biotinylated PCR products. They were performed in triplicate using the PSQ 96MA pyrosequencer (Qiagen, Hilden, Germany), following the manufacturer’s instructions (primers available on request). The level of heterozygosity of each sample was calculated by PSQ 96TMMA allele quantification software (Qiagen), and validated by Sanger sequencing.

### 4.5. Structural In Silico Modeling of C-Terminal Domain of NIPBL Isoforms A and B and Bioinformatic Analysis of the Missense Pathological Variant in Isoform A

Structural in silico analysis of the C-terminal domain of *NIPBL* isoforms A (residues 2541–2804) and B (residues 2541–2697) was performed by using the protein structure prediction system “RaptorX” [12]. From the five putative templates suggested by the system, the one with the best scoring values (*p*-value, Score, uGDT and GDT, uSeqID, and SeqID) was selected and used to build a 3D structure model for the protein C-terminal domain of isoform A.

The pathogenicity of the missense pathological variant was tested with Poly-Phen-2 [13] and PredictSNP [14].

### 4.6. Cell Culture and Colony Formation Assay

Fibroblast cell lines were maintained in a 1:1 mix of RPMI-1640 (R8758, Sigma-Aldrich, Saint Louis, MO, USA) and Ham’s F-10 nutrient mixture (N6908, Sigma-Aldrich), supplemented with 10% FBS (F7524, Sigma-Aldrich), 1:100 dilutions of sodium pyruvate (11360, Gibco, Carlsbad, CA, USA) and 1:200 dilution of penicillin-streptomycin (P4333, Sigma-Aldrich). Cells were maintained for less than 12 passages at 37 °C and 5% CO_2_.

For colony formation assays, cells were seeded in 6-well dishes at the following dilutions: 125, 250, 500, 1000, 2000, and 4000 cells/well. Cells were allowed to attach for 1–3 h and were then irradiated with the indicated doses, using a 137Cs radiation source (IBL 677, CIS Bio international Bedford, MA, USA). Following irradiation, cells were allowed to grow for 12–17 days, washed with PBS, fixed in methanol for 6–9 min, air dried, and stained with a filtered 1:20 dilution of the Giemsa stain (GS500, Sigma-Aldrich).

### 4.7. Quantification of mRNA from NIPBL Isoforms A and B in Patients’ Fibroblasts

The total RNA from the fibroblasts of patients and controls was extracted using the PAXgene Blood RNA Kit (PreAnalaytiX, Hombrechtikon, Switzerland), according to the manufacturer’s instructions. Single-stranded cDNAs were synthesized from 500 ng of RNA, using the First Strand Synthesis Kit (Thermo Fisher, Boston, MA, USA) with random hexamers.

qPCR was performed to quantify both isoforms with the same primers and conditions described in the quantification of tissues. The same procedure was used on *GAPDH* for normalization. The patients´ results were compared to a normal healthy control. The data were log transformed, which made the data normally distributed, and a Student’s t test was performed to compare gene expression data. Differences between experimental groups were considered significant when *p* < 0.05.

## Figures and Tables

**Table 1:** Clinical features of the two patients.

Clinical Data		Patient 1	Patient 2
**Anthropometric data (newborn)**	**Gestational age**	36 weeks	35 weeks
	**Birth weight (g)**	2600 (P10–25)	2420 (P50)
	**Birth length (cm)**	46 (P10–25)	46.7 (P50)
	**Birth OFC (cm)**	31 (P10–25)	30.5 (P10–25)
	**Intrauterine growth retardation**	No	No
**Anthropometric data (last evaluation)**	**Age at evaluation**	14 years 3 month	10 years 8 month
	**Weight (kg)**	54 (P25–50)	36.3 (P25–50)
	**Length (cm)**	159.5 (P25–50)	136.5 (P3–25)
	**OFC (cm)**	52.5 (P3–25)	52.5 (P25–50)
	**Postnatal growth retardation**	No	No
**Limb abnomalities**		Bilateral brachydactyly—clinodactyly 5th finger Bilateral 2–3 syndactyly (feet)	2–3 partial syndactyly (feet)
**Developmental delay**		+	+
**Intellectual disability**		+	+
**Microcephaly**		+	-
**Behavior problems**		+	+
**Atypical wide nose with broad nasal bridge**		+	+
**Hypoacusia**		−	-
**Gastroesophageal reflux disease**		−	+
**Feeding (swallowing) problems**		−	+
**Hirsutism**		+	-
**Cutis marmorata**		−	-

OFC: Occipito-frontal circumference; + present/− not present.

## Supplementary Materials

Supplementary materials can be found at www.mdpi.com/1422-0067/18/3/481/s1.
