# Supplementary material for: mRNA Quantification of NIPBL Isoforms A and B in Adult and Fetal Human Tissues, and a Potentially Pathological Variant Affecting Only Isoform A in Two Patients with Cornelia de Lange Syndrome
Source: Int J Mol Sci. 2017 Feb 23;18(3):481. doi: 10.3390/ijms18030481 (PMC5372497; doi:10.3390/ijms18030481)
Supplement: Supplementary file 1 [file ijms-18-00481-s001.pdf]

Supplementary Materials: mRNA Quantification of *NIPBL* Isoforms A and B in Adult and Fetal Human Tissues, and a Potentially Pathological Variant Affecting Only Isoform A in Two Patients with Cornelia de Lange Syndrome

Beatriz Puisac, María-Esperanza Teresa-Rodrigo, María Hernández-Marcos, Carolina Baquero-Montoya, María-Concepción Gil-Rodríguez, Torkild Visnes, Christopher Bot, Paulino Gómez-Puertas, Frank J. Kaiser, Feliciano J. Ramos, Lena Ström and Juan Pié

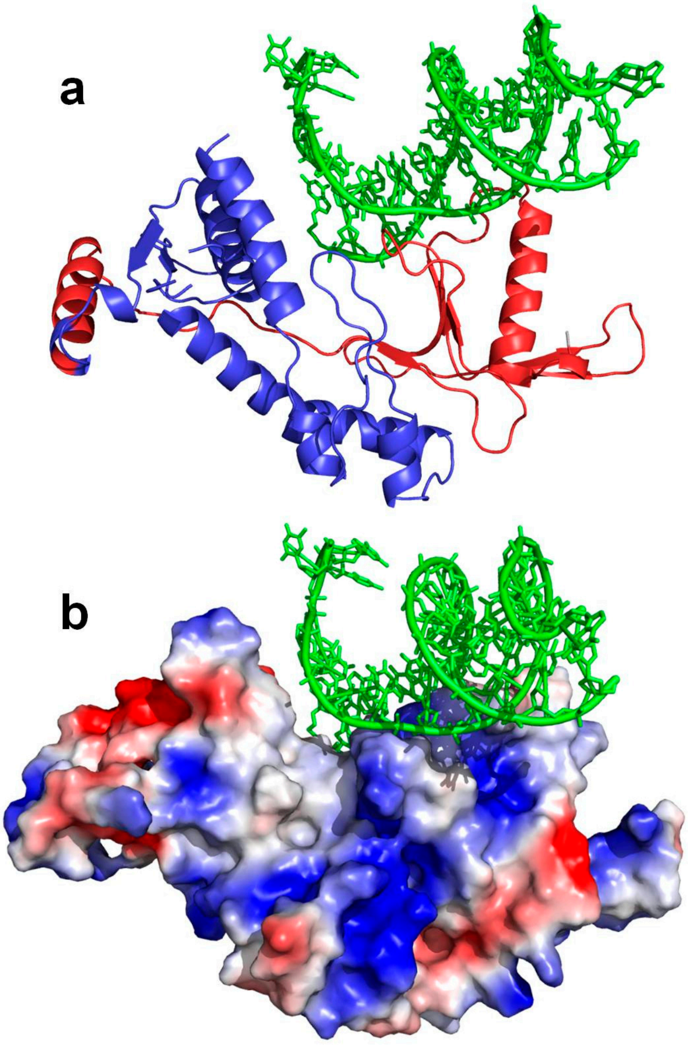

Figure S1. Structural model for the C-terminal domain (residues 2541-2804) of NIPBL isoform A, as obtained from the RaptorX server (http://raptorx.uchicago.edu/). (**a**) 3D model showing secondary structure elements. Blue: residues 2541-2682, shared by both isoforms A and B. Red: residues 2683-2804, present in isoform A and absent in isoform B. Green: putative position of DNA molecule, according to the structure template (Protein Data Bank entry = 3kd1E). Note that most protein-DNA contacts are located in the protein domain absent in NIPBL isoform B. (**b**) Model surface, colored according to electrostatic charge (blue: positive, red: negative), showing positive patches in contact to the negatively charged DNA molecule.
